# Good cop, bad cop? Rethinking the roles of cardiac macrophages in injury

**DOI:** 10.1172/JCI208767

**Published:** 2026-08-03

**Authors:** Janmes Karunamurthy, Ziyi Li, Ajitha Thanabalasuriar

**Affiliations:** 1 Department of Pharmacology and Therapeutics and; 2 Department of Microbiology and Immunology, McGill University, Montreal, Quebec, Canada.

## Abstract

Cardiac macrophages (CMs) preserve homeostasis in the heart by clearing cellular debris and facilitating electrical conduction. During tissue injury, embryonically derived CMs (em-CMs) have traditionally been deemed beneficial for promoting tissue repair, whereas monocyte-derived CMs (mo-CMs) are considered detrimental, contributing to inflammation and tissue damage. However, Kasam et al. challenge this binary classification using cardiac-specific strategies to expand either em-CM or mo-CM populations. As expected, mice with cardiac-specific mo-CM expansion exhibited adverse outcomes following transverse aortic constriction (TAC). Surprisingly, mice with expanded em-CMs also showed a marked decline in cardiac function after TAC, which was associated with an unexpected interaction with mo-CMs. This deterioration was temporally regulated, occurring only if em-CMs were expanded before TAC induction. Together, these findings suggest that simplistic classification of CMs as either beneficial or harmful underestimates their complex roles in cardiac pathology, highlighting the need to reassess current views of macrophage function in heart injury.

## Introduction

The heart is the most tireless muscle in the human body, beating the entirety of our lifetime. Among the major cell types that make up the heart, such as cardiomyocytes and epicardial cells, resides a finely tuned resident immune system that maintains homeostasis and responds to injuries or diseases. At steady state, the most abundant immune cells in the heart are tissue-resident cardiac macrophages (CMs) (1). CMs consist of two broad lineages, embryonically derived (em-CMs) and monocyte-derived macrophages (mo-CMs), both dispersed throughout the heart tissue (2, 3).

Em-CMs form the main myeloid population in the adult heart. Em-CMs persist through self-renewal and can be characterized by their expression of CX3CR1 and overall antiinflammatory phenotype (2, 4, 5). During homeostasis, em-CMs have been shown to directly facilitate cardiac electrical conduction and guide early lymphatic development (6, 7). In contrast, mo-CMs only form a small population in the adult heart at steady state (2, 8). Mo-CMs are distinguished by their retained expression of CCR2 after differentiation from monocytes, alongside being predominantly pro-inflammatory (5, 8, 9). Mo-CMs play little to no role during homeostasis but are recruited in copious amounts upon heart injury (5, 10).

Studies in previous cardiac infract models have indicated that distinct CM lineages differentially contribute to healing process of the heart. While em-CMs resolve infarct injury and reduce cardiac dysfunction, mo-CMs exacerbate the tissue injury and cause functional decline of the heart (2, 8). However, is biology ever this simple? Can one cell lineage always be the hero in every story? The study by Kasam et al. in this issue of the *JCI* reveals that the current classification of heart macrophage populations is not so clearly defined as assumed. They demonstrate that expansion of em-CMs prior to cardiac injury may inadvertently elicit activation of mo-CMs that contributes to declining heart functions (11).

## The different shades of macrophages and heart disease

Previous studies have used combinations of systemic depletion methods and mouse models of injury to understand the importance of CMs in heart tissue repair. Among injury models, ischemic infarct and transverse aortic constriction–induced (TAC-induced) pressure overload are commonly used to understand acute or chronic cardiac injury, respectively (12). As hypertension is a leading factor in development of heart disease, the use of TAC is pertinent in obtaining translatable findings for understanding heart failure (13).

Existing systemic CM depletion studies using murine models show reparative roles for em-CMs, whereas mo-CMs are predominantly inflammatory and pro-fibrotic (2, 4, 14–17). TAC stimulation in mice with antibody-mediated ablation of em-CMs increased fibrosis, reduced microvascular density, and worsened ejection fraction (14). Similarly, diphtheria toxin depletion of CX3CR1^+^ em-CMs reduced ventricular systolic function and increased mortality in ischemic infarction (2). Meanwhile, pharmacological blockade of CCR2^+^ mo-CMs following TAC both improved ejection fraction and reduced fibrosis at 4 weeks post-TAC (15). Together, these results suggest clear roles for em- and mo-CMs in models of heart disease, while revealing their potential as therapeutic targets (5, 10).

To bridge results in mice to a human context, prior studies have been conducted on human explants from biopsies. While human tissue samples are limited to small regions of the heart, such as the left and right ventricular walls, these studies have broadly corroborated the results seen in mice regarding mo- and em-CMs across species. Human biopsy work reinforces the interpretation that the human heart contains distinct mo- and em-CM lineages, which contribute differentially to injury (18).

## Breaking the dogma

While earlier studies have revealed many details of CMs and their lineages, one major caveat of their results is the use of systemic models (5, 8, 10). Whole-body genetic knockouts and broad pharmacological inhibition are useful research tools, but it is difficult to conclude the results are specific to the cells of interest. Thus, understanding the organ-specific consequences of targeting CMs is required to establish their needed medicinal potential. In this issue of the *JCI*, Kasam et al. bring clarity to this knowledge gap by utilizing a cardiac-specific gain-of-function approach (11).

Using viral vectors encoding CC chemokine ligand 2 (*Ccl2*) or colony-stimulating factor-1 (*Csf1*) under the control of the cardiac troponin promoter, the authors elevated cardiac levels of CCL2 and CSF1 in mice. This led to heart-specific expansion of CCR2^+^ mo-CMs and CX3CR1^+^ em-CM populations, respectively. With this approach, Kasam et al. showed that increased numbers of CCR2^+^ or CX3CR1^+^ CMs in the heart did not alter baseline cardiac function, which was evaluated for over 1 year after initial stimulation in young and aged animals. Curiously, in aged animals, elevated CSF1 levels promoted proliferation of em-CMs, challenging the prevailing view that these cells lose their replicative capacity with age (19). Additionally, expansion of CCR2^+^ macrophages in adult mice resulted in a corresponding reduction in a subset of CX3CR1^+^ macrophages, suggesting competition for shared niches between these populations.

**Figure 1:**
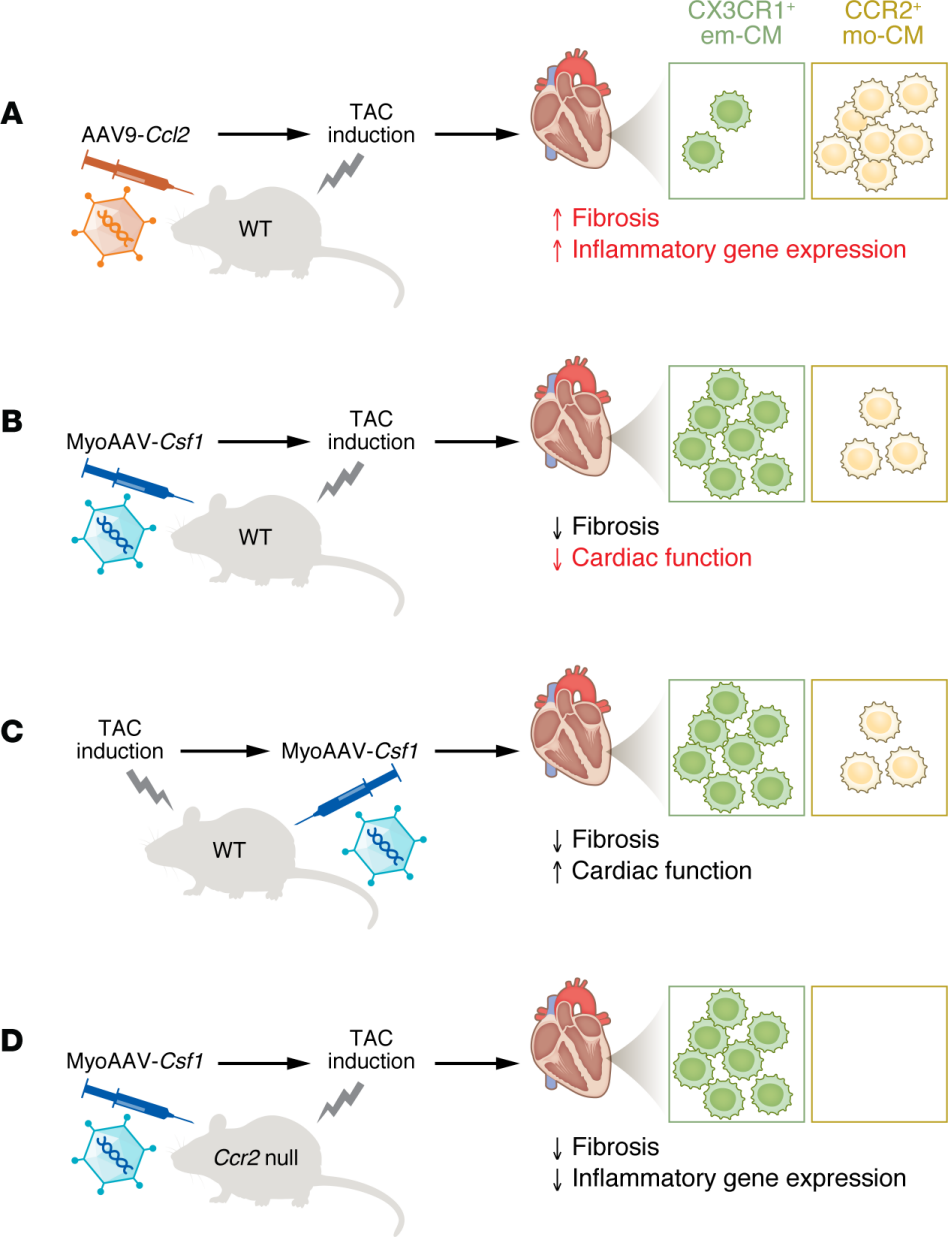
Cardiac macrophages alter cardiac stress responses. Kasam et al. used cardiac-specific strategies to selectively expand either mo-CM or em-CM populations in the context of experimental TAC (11). (**A**) When treated with AAV9-*Ccl2*, expansion of CCR2^+^ mo-CMs increased both fibrosis and inflammatory genes after TAC induction. (**B**) Mice given MyoAAV-*Csf1* before TAC was induced had elevated CX3CR1^+^ em-CMs and faced a significant decline in heart function alongside a reduced fibrotic response. (**C**) Wild-type mice challenged with TAC prior to MyoAAV-*Csf1* treatment had preserved cardiac function compared with **B**, with similar decrease in fibrosis observed. (**D**) *Ccr2*-null mice were administered MyoAAV-*Csf1*, producing high numbers of only em-CMs in the heart. After TAC stimulation, these mice showed reduced fibrosis and reduced inflammatory gene expression and no decline in heart function compared with controls or the MyoAAV-*Csf1*–treated mice in **B**.

To understand the role of each CM lineage in heart failure, the authors examined the responses of treated mice to TAC-induced pressure overload. Elevated cardiac CCL2 levels amplified inflammatory cytokine production and exacerbated fibrosis 8 weeks after TAC, consistent with the established pathogenic role of CCR2^+^ mo-CMs (Figure 1A). However, no hypertrophy or decline of systolic function was observed. Yet, TAC in animals with expanded CX3CR1^+^ em-CMs did cause severe reductions in systolic pumping ability despite no increase in fibrosis relative to the control (Figure 1B). Interestingly, when TAC was performed prior to elevation of CX3CR1^+^ CMs, no significant changes in cardiac function or fibrosis were observed (Figure 1C). These findings suggest that CX3CR1^+^ CMs can play both protective and detrimental roles in early heart failure, depending on the timing of their expansion.

Kasam et al. went on to show compelling mechanistic insights by examining the potential pathological role of CCR2^+^ CMs in em-CM expanded hearts after TAC induction. In animals lacking CCR2^+^ CMs, elevated cardiac *Csf1* expression followed by TAC no longer produced the previously observed decline in cardiac function. Instead, these mice exhibited a reduced inflammatory profile by 8 weeks post-TAC while maintaining the low-fibrosis phenotype as seen in mice with high cardiac *Csf1* alone (Figure 1D). These results suggest that although CCR2^+^ mo-CMs are key drivers of adverse outcomes in heart failure, the CX3CR1^+^ CM population may play a role in coordinating the pro-inflammatory effects through communications with CCR2^+^ CMs.

## Conclusion

Cardiovascular diseases are the single leading cause of death globally (13, 20). To sufficiently address this public health issue, new and more effective therapies are needed. Pharmacologically targeting CMs is an attractive concept currently under investigation, with promising preclinical results (2, 16, 17). Kasam et al. have revealed a potentially novel communication pathway between em-CM and mo-CMs that results in adverse effects on heart function. Future studies aimed at elucidating the functional roles and consequences of interactions between em-CMs and other cell types will be essential for their development as a therapeutic target.

A subsequent question that lingers after Kasam et al.’s work is, How exactly do high numbers of em-CMs elicit mo-CM release of pro-inflammatory mediators upon TAC? The heterogeneity and cellular subsets of em-CMs may be one possibility. Specific subpopulations of em-CMs may be responsible for mediating the pro-inflammatory communication pathway with mo-EMs (2). Transcriptomics have shown subsets of em-CMs, such as clusters of cells highly expressing interferon-stimulated genes, that remain unstudied in sterile inflammation (2, 21). Perhaps targeting a subset of em-CMs is required to prevent decline in heart function after TAC. Additionally, future studies to investigate why the timing of em-CM expansion is crucial to their beneficial or harmful effect are needed. Understanding the details of mo-CM to em-CM crosstalk during the critical window of em-CM expansion is required to take full advantage of the reparative potential of CMs.

Beyond treatment of heart disease, the authors present tantalizing data on the ability to expand em-CMs in aged animals. Age-associated cardiac decline is a prominent issue that has been linked to increased inflammatory mediators in the heart (22). In this context, with age decreasing the number of em-CMs, being able to restore this antiinflammatory population could be a future therapeutic opportunity (19).

Kasam et al.’s findings also call for a reevaluation of comparable macrophage lineages observed in other organs. Macrophages in similar embryonically and monocyte-derived niches are known in the interstitial spaces of the lung and skin, so could they be similarly more nuanced than previously thought (9)? While it remains unclear how to adapt CMs into a long-term therapeutic, the study by Kasam et al. provided key insights for the next steps to understanding macrophages in the heart and beyond.

## Conflict of interest

The authors have declared that no conflict of interest exists.

## Funding support

- AT by the Canadian Institute of Health Research (CIHR, fund number: 202209PJT-487169-IMN-CFAA-180756) and Natural Sciences and Engineering Research Council of Canada (fund number: DGECR-2022-00172).
- JK by the CIHR Canada Graduate Research Scholarship - Master’s program.
- ZL by the China Scholarship Council - Doctorate Training.

## Version 1 08/03/2026

Electronic publication
